# In situ disordering of monoclinic titanium monoxide Ti_5_O_5_ studied by transmission electron microscope TEM

**DOI:** 10.1038/s41598-017-11164-6

**Published:** 2017-09-07

**Authors:** А. А. Rempel, W. Van Renterghem, А. А. Valeeva, M. Verwerft, S. Van den Berghe

**Affiliations:** 1 Institute of Solid State Chemistry, Ural Branch, Russian Academy of Sciences, Pervomaiskaya 91, Ekaterinburg, 620990 Russia; 2 Ural Federal University, Mira 19, Ekaterinburg, 620002 Russia; 3 SCK-CEN, Boeretang 200, Mol, 2400 Belgium

## Abstract

The superlattice and domain structures exhibited by ordered titanium monoxide Ti_5_O_5_ are disrupted by low energy electron beam irradiation. The effect is attributed to the disordering of the oxygen and titanium sublattices. This disordering is caused by the displacement of both oxygen and titanium atoms by the incident electrons and results in a phase transformation of the monoclinic phase Ti_5_O_5_ into cubic *B*1 titanium monoxide. In order to determine the energies required for the displacement of titanium or oxygen atoms, i.e. threshold displacement energies, a systematic study of the disappearance of superstructure reflections with increasing electron energy and electron bombardment dose has been performed *in situ* in a transmission electron microscope (TEM). An incident electron energy threshold between 120 and 140 keV has been observed. This threshold can be ascribed to the displacements of titanium atoms with 4 as well as with 5 oxygen atoms as nearest neighbors. The displacement threshold energy of titanium atoms in Ti_5_O_5_ corresponding with the observed incident electron threshold energy lies between 6.0 and 7.5 eV. This surprisingly low value can be explained by the presence of either one or two vacant oxygen lattice sites in the nearest neighbors of all titanium atoms.

## Introduction

The study of the vacancy defect structure and the order-disorder phase transformations in titanium oxides of different stoichiometry draw the increasing attention as their importance for synthesis of the materials with the distinct functional optical or electronic properties. Additionally, materials based on titanium oxides are very promising for purification of water because of their high photocatalytic activity and for hydrogen industry for splitting of water into hydrogen and oxygen, etc.

The low- and high-temperature stability of the crystal structure (space group *C*2/*m*) of titanium monoxide Ti_5_O_5_ with a very high number of vacant crystal lattice sites^1^ is of pivotal interest. The structure of Ti_5_O_5_ consists of two equal monoclinic sublattices shifted by the [½ ½ 0] vector of the *C*2/*m* lattice. At high temperatures, above 1223 K, both sublattices are disordered and the *B*1 structure (space group *Fm*-3*m*), with the same amount of vacant lattice sites of about 16.7 at.% becomes stable^1^.

Analysis of short-range order in Ti_5_O_5_ shows that, depending on the crystallographic position, 1 or 2 sites of the 6 lattice sites in the first oxygen coordination sphere of the titanium sublattice_,_ and 2 or 3 sites of the 12 lattice sites in the first titanium coordination sphere of the titanium sublattice are not occupied^2^. The same low occupancy of lattice sites is found around each oxygen atom in titanium monoxide. In spite of low coordination number and large amount of vacancies, the ordered Ti_5_O_5_ phase is stable in a wide temperature range from low temperatures up to 1223 K.

The low coordination numbers of Ti_5_O_5_ imply that each atom can be removed from its position much more easily than in a close-packed structure. Indeed, the superlattice reflections of TiO_*x*_, caused by the ordering of atomic-vacancy ordering, can be effectively removed under electron irradiation at 200 keV^3^. So, the *in situ* observation of the displacement of atoms in TEM, operating at lower voltages, is possible similar to the experiments of Venables *et al*. on nonstoichiometric vanadium carbide^4^. In order to determine the displacement threshold energy in Ti_5_O_5_, a systematic irradiation study of the ordered monoxide in a wide range of incident electron energies less than 200 keV and the *in situ* observation of the irradiated area have been performed in this work. The data on threshold energies will give access to the strength of chemical bonds and clarify the nature of the remarkable thermal stability of the vacancy rich binary compound Ti_5_O_5_.

## Results and Discussion

**Figure 1:**
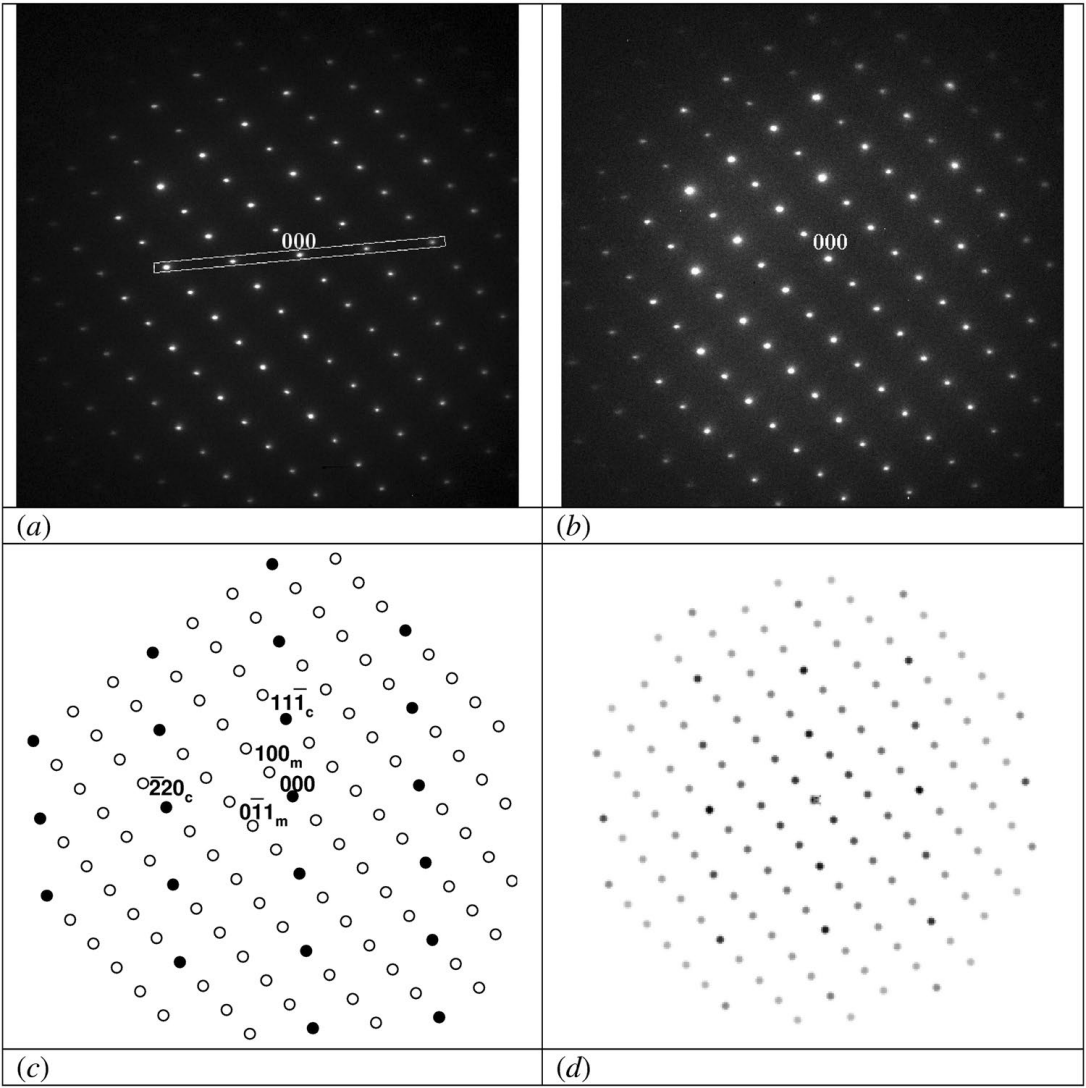
Nondisappearing of superstructural reflections of (0-1-1) monoclinic zone after irradiation at 100 keV at the beginning (**a**) and after long-term irradiation (**b**). The line strip, which was used for the evaluation of the intensity of superstractural reflections, is shown additionally in (**a**). (**c**) Schematic representation of the zone with structure reflections (close symbols) and superstructure reflections (open symbols) together with important Miller indices for cubic zone (-1-1-2) (subscript c) and monoclinic (0-1-1) zone (subscript m); (**d**) simulated electron diffraction image for the case of disordered oxygen and ordered titanium sublattice.

**Figure 2:**
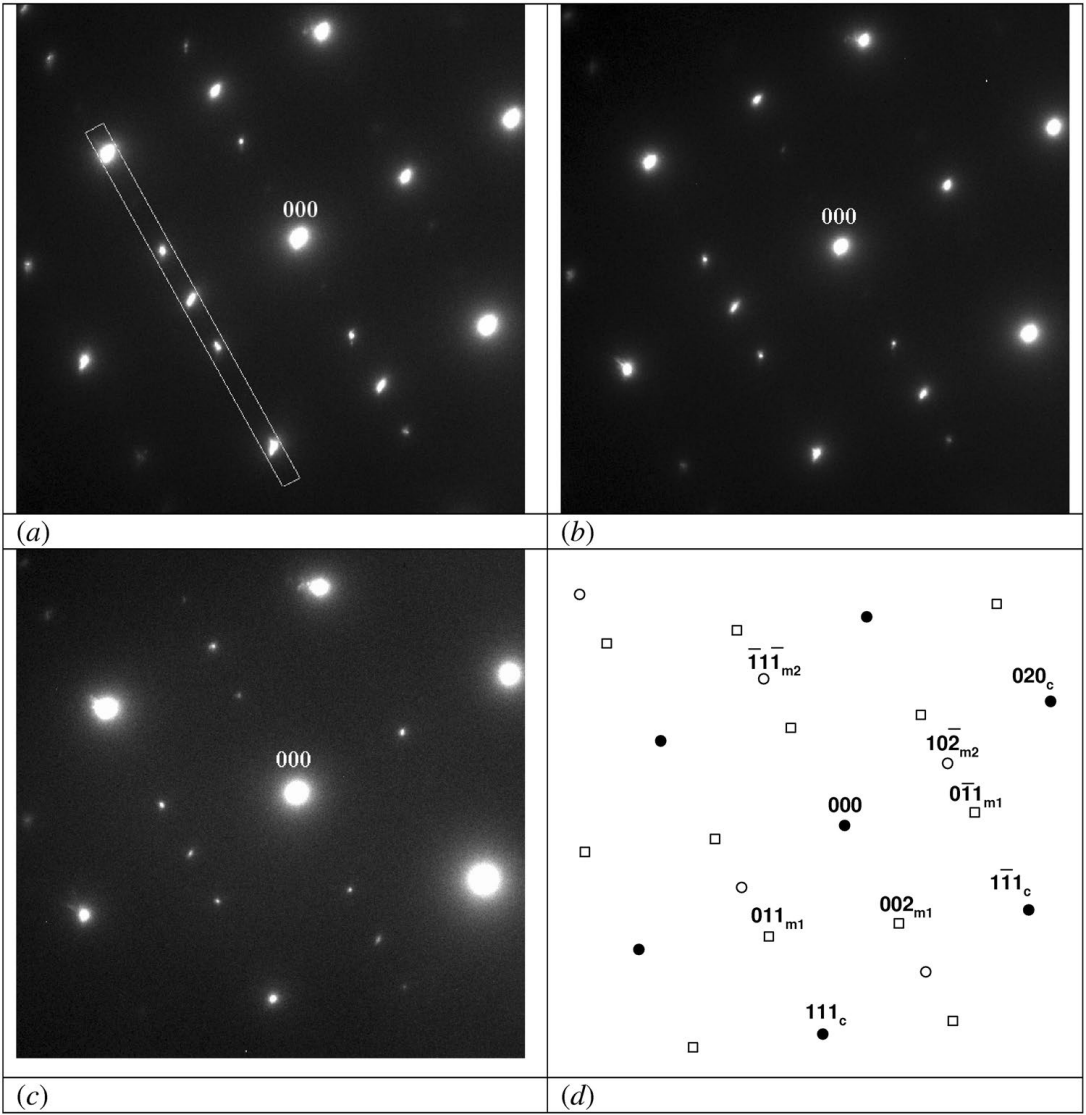
Slowly disappearing superstructural reflections of two monoclinic domains (100) and (231) after irradiation at 120 keV. (**a**) at the beginning of irradiation; (**b**) after 40 min of irradiation (dose of 1.87·10^24^ m^−2^); (**c**) after 80 min of irradiation (dose of 3.75·10^24^ m^−2^); (**d**) structure (closed symbols) and superstructure (open symbols) spots in a schematic representation. Miller indices for cubic zone (10-1) (subscript c) and two monoclinic zones (100) (subscript m1) and (231) (subscript m2) are shown additionally.

**Figure 3:**
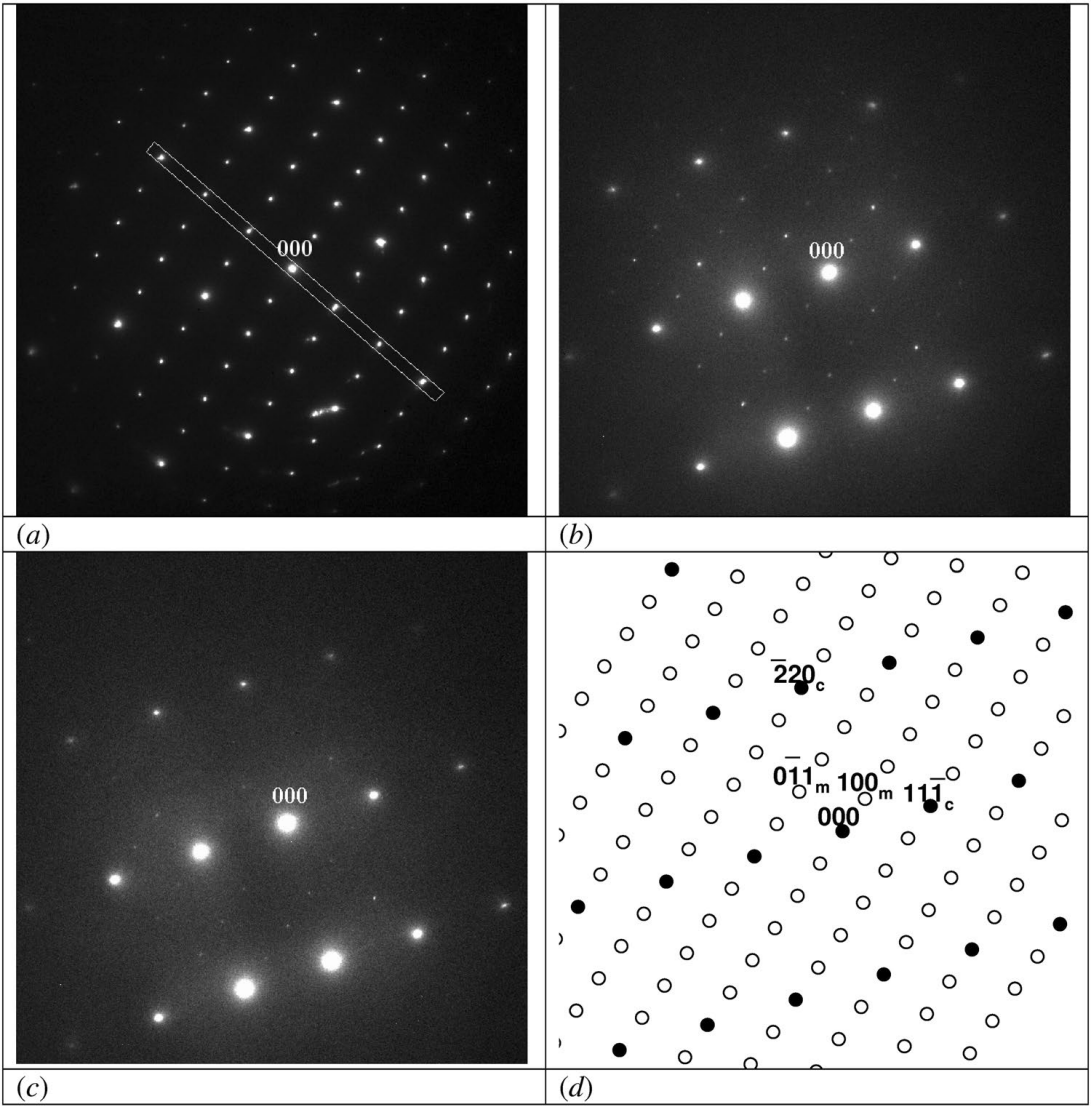
Fast disappearing of superstructural reflections of (0-1-1) monoclinic zone after irradiation at 140 keV. (**a**) at the beginning of irradiation; (**b**) after 20 min of irradiation (dose of 0.55·10^24^ m^−2^); (**c**) after 40 min of irradiation (dose of 1.1·10^24^ m^−2^); (**d**) structure (closed symbols) and superstructure (open symbols) spots in a schematic representation. Miller indices for cubic zone (-1-1-2) (subscript c) and monoclinic (0-1-1) zone (subscript m) are shown additionally.

The electron diffraction patterns are shown in Figs 1, 2, and 3. The intensity of the pattern was adjusted such that the camera was never saturated. The response curve of the CCD camera is linear up to saturation. Therefore, the measured intensity of a diffraction spot on the digital image is directly proportional to the number of diffracted electrons, which is in the kinematic approximation proportional to the volume fraction of each phase. The intensity of every spot was measured by integration over the whole area of spot. Because of the non-spherical shape of Ti_5_O_5_ ordered nano-domains some spots are not circular and have ellipsoidal shape, so the used integration allowed determining the intensity independently of the shape of spot.

For each pattern at the same accelerating voltage, the same recording time was used. For each different accelerating voltage a fresh, i.e. not yet irradiated, particle was chosen. The cubic zone indices for the different irradiations are given in Table 1.

**Table 1:** Parameters of the electron irradiation conditions in TEM. The maximum energy transferred to the titanium and oxygen atoms *E*_T,max_, and the type of cubic zones are also given.

Incident electron energy *E*_e_, keV	Maximum transferred energy *E*_T, max_, eV		Electron flux *J*, 10^20^ m^−2^∙s^−1^	Maximum time of irradiation *t*_max_, min	Cubic zone indices [*hkl*]	Have the superstructure reflections disappeared?
	Ti	O				
100	5.03	15.1	5.27	140	112	No
110	5.59	16.7	5.36	140	113	No
120	6.15	18.4	7.81	80	110	Slow disappearing
130	6.72	20.1	9.77	80	110	Slow disappearing
140	7.30	21.8	4.57	60	112	Fast disappearing
180	9.71	29.0	7.81	40	112	Yes
200	11.0	32.8	9.26	47	110	Yes

Because of the higher symmetry of the disordered structure, the ordered structure fits the disordered structure in different ways. For Ti_5_O_5_, 12 differently oriented monoclinic domains can be formed. Even after long-term annealing of the specimen, the lateral dimensions of these domains remain small and often several domains contribute to one diffraction pattern, even if the smallest selected area aperture is used. For example, for the irradiations at 120 keV (Fig. 2) and 200 keV, areas with at least two domains were used and for the irradiations at 110 and 180 keV, areas with at least three domains were explored.

**Figure 4:**
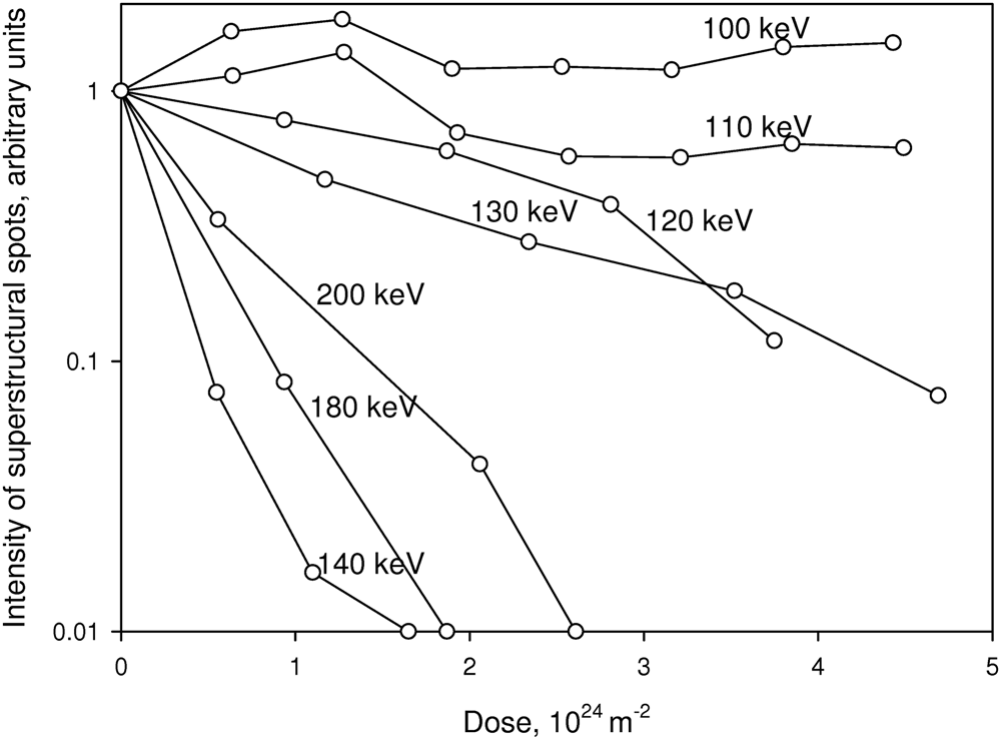
Normalized intensities of superstructural spots of Ti_5_O_5_ phase for irradiations with different electron energies (100, 110, 120, 130, 140, 180, 200 keV) in dependence of the dose of electron irradiation.

To measure the intensity of one row of reflections in the diffraction pattern a strip is placed over each row of reflections with a width slightly larger than the widest spot in the row (see examples in Figs 1a, 2a, 3a). The intensity is averaged over the width of the strip and from the intensity profile, the intensity of each reflection, after subtraction of the background, is measured. The ratio between the accumulated intensity of all reflections belonging uniquely to the domains of the monoclinic phase *C*2/*m* and the accumulated intensity of the *B*1 type reflections has been calculated. This ratio does not give the exact value of long range order in the ordered phase Ti_5_O_5_, but the disordering under irradiation can be monitored. The ratio will decrease when the structure becomes more disordered. Moreover, all ratios have been normalized to the value before irradiation to compensate for the difference in results between the different orientations of the grains used for the measurements at different voltages. The results for all irradiations are presented in this form in Fig. 4.

In the diffraction patterns, it has been observed that the superstructure reflections and domain structures characteristic for ordered Ti_5_O_5_ disappear after irradiation with electrons with energies equal to and higher than 120 keV (Table 1 and Fig. 4). Superstructure reflections disappeared independently of the orientation of the crystal to the electron beam (see Table 1). Only structural reflections characteristic for disordered titanium monoxide TiO_*y*_ with a *B*1 structure were observed after irradiation with electrons with energies higher than 140 keV. No diffuse scattering, characteristic for short range order^4^ or clustering of vacancies^5, 6^, was observed in the diffraction patterns after irradiation.

The superstructure reflections do not disappear after irradiation at 100 and 110 keV. Even after 140 min, corresponding with a dose of 4.5∙10^24^ m^−2^, all reflections still have approximately the same intensity as before radiation. Figure 1a and b show examples for the monoclinic[0-1-1] zone before and after irradiation with 100 keV electrons. In Fig. 2 it is shown that after irradiation at 120 keV the superstructure reflections belonging to at least two domains of the ordered phase, i.e. the^100^ and the^231^ zones, disappear. Figure 3 shows the disappearance of the superstructure reflections of the monoclinic[0-1-1] zone after irradiation at 140 keV. In Figs 1c, 2d, and 3d the schematic representation of structural and superstructural reflections is given to clarify the diffraction pattern.

Figure 4 shows the behavior of the relative intensities of superstructure reflections with the dose on a logarithmic scale. The intensity *I* of a reflection is proportional to the square of the structure factor. The structure factor is proportional to the long range order parameter, which is inversely proportional to the flux *J* of incident electrons^4, 7^

$${\rm{d}}\,l{\rm{n}}\,I/{\rm{d}}t=-k{\sigma }_{{\rm{d}}}\cdot J,$$

where σ_d_ is the cross section for the displacement of an atom by an incident electron; *k* is a constant depending on the type of ordered structure, the crystallographic positions of the displaced atoms and the amount of multiple collisions (cascade effects). So, the slope of the graphs in Fig. 4 should be proportional to the displacement cross section.

The number of displaced atoms is proportional to the dose and the displacement cross-section of the atoms. The displacement cross-section for Ti atoms increases if the incident electron energy increases from 120 to 200 keV^4^. Therefore, above the displacement threshold, the number of displaced atoms should increase for the same dose and the slopes of the curves for the higher energies should be larger. In Fig. 4 it is clearly seen, that there are at least two groups of lines: 100, 110 keV with no disappearing superstructure reflections, and 120–200 keV with decreasing intensity of superstructure reflections. The fact that the curve for 140 keV has a higher slope than the curve for 180 and 200 keV is not clear and can be a consequence of measurement uncertainties.

The atomic scattering factor of titanium atoms is much higher than that of oxygen atoms. Therefore, the intensity in the diffraction patterns is mainly determined by the distribution of titanium atoms in titanium monoxide and not by oxygen atoms. Modeling of diffraction images shows that, in case of an ordered titanium sublattice, the intensities of superstructure reflections do not change within experimental uncertainties if the oxygen sublattice undergoes disordering (see Fig. 1d). Therefore, the fact that superstructure reflections do not disappear at irradiation at 110 keV, but do above 120–140 keV, means that the energy of 120–140 keV corresponds to the lowest energy required for the disordering of the titanium sublattice. A simple explanation for this disordering could be the direct displacement of titanium atoms at 120–140 keV incident electron energy.

Because elastic collisions between the incident electrons and atoms satisfy the relativistic Rutherford scattering relations to a good approximation^8^ the maximum energy an atom with a mass *M* can get from an electron with energy *E*_e_ is equal to

$${E}_{{\rm{T}},{\rm{m}}{\rm{a}}{\rm{x}}}=(4{m}_{{\rm{e}}}/M)(1+{E}_{{\rm{e}}}/2{m}_{{\rm{e}}}{c}^{2}){E}_{{\rm{e}}},$$

where *m*_e_ is the electron rest mass and *c* the velocity of light. So, the maximum energy which will be transferred to a titanium atom at 120 and 140 keV equals 6.15 and 7.30 eV respectively. Therefore, the displacement threshold for titanium atoms in titanium monoxide is between about 6.0 and 7.5 eV. This displacement threshold is the lowest one ever found for metal atoms in solids (see Table 2).

**Table 2:** Threshold energies for the displacement of metal M and nonmetal X atoms in Ti_5_O_5_ and in similar substances.

Substance	Threshold energy *T*_d_, eV		Incident electron energy *E*, keV	Direct measurements or estimation	Reference
	M-metal	X-nonmetal			
Ti_5_O_5_	5.59–7.30		120–140	Direct	This work
Ti_5_O_5_	—	5.59–7.30	40–52	Estimation	This work
TiO_x_	—	—	<200	Estimation	3
Ti	22.3 ± 0.3		360 ± 10	Direct	9
Ti_2_C		≪20	<100	Estimation	13
(Ti, Mo)C_*y*_	<270		<2000	Estimation	14
V_8_C_7_	—	2.8 ± 0.3	125	Estimation	15
V_6_C_5_	—	5.4	<33	Estimation	4
TaC_0.99_	—	23.2 ± 1.1	115	Direct	10
TaC_0.99_	42 ± 2	28 ± 6	≈1500	Direct	11
TaC_0.80_	32 ± 2	28 ± 6	<350	Direct	12
ZrN_x_	—	<17.2	200	Direct	13

In ordered titanium monoxide there are two different types of titanium positions, one with 4 titanium-oxygen nearest neighbours bonds (20% of titanium atoms) and one with 5 (80% of titanium atoms). It is logical to attribute the observed threshold energy to titanium atoms sitting in positions either with the 4 or 5 bonds. In this case the energy required to break one titanium-oxygen bond should be about 1.5 eV.

Even if it is not possible to see the structure of the oxygen sublattice by electron diffraction, from the data obtained for displacement threshold energies of titanium atoms one can estimate the incident electron energy required for the displacement of oxygen atoms. The structure of titanium monoxide is symmetrical with respect to the replacement of a Ti atom by an oxygen atom and vice versa. Taking also into account that the bonding energy comes mostly from only first neighbours, one can estimate that the energy which should be transferred to an oxygen atom in order to displace it, is roughly the same as for titanium atom, i.e. between 6.0 and 7.5 eV depending on the type of oxygen position. Because of the lower mass of an oxygen atom, this energy range corresponds to incident electron energy between 42 and 52 keV. The electron energies used in this work are in all cases much higher than this energy and consequently the oxygen sublattice will be disordered during each irradiation experiment.

The fact that no effect of irradiation is seen below 110 keV (Table 2, Fig. 4) means that the titanium sublattice remains ordered at ambient temperature in the presence of the disordered oxygen sublattice. Additional studies are required to confirm this statement and to deduce the state of oxygen sublattice, dynamical or static disorder, during irradiation or, static order or static disorder, after irradiation is finished.

Very few direct measurements^7, 9–12^ and some estimation from experimental data^4, 13–15^ of displacement thresholds are available at present. The literature data for the threshold energies of Ti displacement and of metal and non-metal atoms in compounds similar to titanium monoxide are given in Table 2. From this table it can be seen very clearly that the threshold displacement energy for titanium atoms in titanium monoxide is extremely low in comparison to this energy for titanium and other metal atoms in similar compounds. The threshold displacement energy of titanium atoms in titanium monoxide is close to the low value found for carbon atoms in carbon vacancy rich ordered vanadium carbide V_6_C_5_^4^.

In case of close-packed metals or compounds, the displacement threshold energy has a contribution from the bonding energies as well as from the required local expansion of the lattice in order to push an atom into an interstitial position through neighbouring atoms. In titanium monoxide Ti_5_O_5_, each titanium atom has at least one vacancy in the first titanium coordination sphere. Similarly, in vanadium carbide V_6_C_5_, which has comparable displacement threshold energy for carbon, each carbon atom has one vacancy in the first carbon coordination sphere. This suggests that the low displacement energies are directly connected to the bonding energies of atoms in these compounds. Indeed, for the displacements of atoms no high energy interstitials should be produced by irradiation. So, in case of high vacancy concentrated substances the bonding energies can be calculated as the division of the threshold displacement energy by a number of bonds. As it was shown above in case of titanium monoxide one can deduce that the Ti-O bond energy is about 1.5 eV.

## Conclusion

The disordering of the monoclinic Ti_5_O_5_ phase under electron beam irradiation has been studied with TEM. A threshold has been found between 120 and 140 keV. This incident electron energy interval corresponds to threshold displacement energy between 6.0 and 7.5 eV per Ti atom and is attributed to the displacements of titanium atoms. From the determined displacement threshold energies, titanium-oxygen bond energy about 1.5 eV is deduced for this compound. This value should be useful for understanding the stability of Ti_5_O_5_, activation energies for diffusion and order-disorder phenomena observed earlier in this compound. This value is also useful for understanding the stability of defect structures in nonstoichiometric TiO_2-x_, which is recently found to be very promising material for environmental purposes and hydrogen industry because of their high photocatalytic activity under sun light^16–19^ and photocatalytic splitting of water into hydrogen and oxygen^20^. The vacancy structure and relatively low stability of Ti_5_O_5_ under electron beam will be useful to understand formation of structural vacancy free titanium monoxide polymorphs under high pressure^21^ and ε-TiO recently found in^22^.

## Methods

Ordered monoclinic Ti_5_O_5_ was prepared by long term annealing of high temperature sintered polycrystalline titanium monoxide^23^. After preheating the sintered specimen at a temperature of 1330 K for 4 hours, the temperature is slowly decreased to ambient temperature at a rate of 10 K/h in a sealed evacuated quartz tube. After applying this heat treatment, a maximum atomic-vacancy order has been achieved.

By chemical analysis (for details see ref. 24) it was shown that the atomic ratio O/Ti is equal to 0.99 which is close to the stoichiometric composition of the Ti_5_O_5_ phase. Full profile Rietveld-like analysis of the X-ray powder diffraction pattern showed that the content of the Ti_5_O_5_ phase is close to 100% and the lattice parameters of this phase with space group *C*2/*m* (*A*12/*m*1) are: *a* = 586.2; *b* = 414.3; *c* = 934.2 pm; and *β* = 107.5° (for details see ref. 24).

For the TEM study, the polycrystal was crushed down to micrometer size particles which were deposited on a Cu grid. The thinnest particles protruding from the grid bars are used for the TEM analysis.

The specimens have been irradiated at room temperature in a JEOL 3010 TEM at accelerating voltages of 100, 110, 120, 130, 140, 180 and 200 kV. Values for the electron flux and dose have been estimated, based on the electron current density measured on the fluorescent screen. The typical electron fluxes ranged from 5∙10^20^ m^−2^∙s^−1^ to 1∙10^21^ m^−2^∙s^−1^. The dose was calculated by multiplying the irradiation flux with exposure time.

The electron diffraction patterns have been recorded with a Gatan slow scan digital camera. The intensity of the pattern was adjusted such that the camera was never saturated. For each pattern at the same accelerating voltage, the same recording time was used. For each different accelerating voltage a fresh, i.e. not yet irradiated, particle was chosen. The intensity of one row of reflections in the diffraction pattern was measured using Digital Micrograph^®^. Because, in many cases the diffraction spots are not circular, to estimate the intensity value the integration over a width covering the entire diffraction spot was performed. Then the intensity over the length of the spot was measured and the background intensity was subtracted.
